# The impact of FFP3 respirators on the blood saturation

**DOI:** 10.1038/s41598-022-05319-3

**Published:** 2022-01-25

**Authors:** Izabela Wojtasz, Krystyna Jaracz, Paweł Sobczyński, Artur Drużdż, Danuta Dyk, Radosław Kaźmierski

**Affiliations:** 1 Institute of Health Sciences, Collegium Medicum, University of Zielona Gora, 65-417 Zielona Gora, Poland; 2 Department for Neurology with Stroke Unit, L. Bierkowski Hospital, Poznan, Poland; 3 Department of Neurological Nursing, Faculty of Health Sciences, Poznan University of Medical Sciences, 60-179 Poznań, Poland; 4 Department of Anesthesiology and Intensive Therapy, Poznan University of Medical Sciences, 61-848 Poznań, Poland; 5 Department of Neurology, Municipal Hospital in Poznań, Poznań, Poland; 6 The Institute of Anesthesiological and Intensive Care Nursing, Faculty of Health Sciences, Poznan University of Medical Sciences, Poznan, Poland; 7 Department of Neurology, University of Zielona Gora, 65-046 Zielona Gora, Poland; 8 Department for Neurology, Poznan University of Medical Sciences, 61-701 Poznan, Poland

**Keywords:** Occupational health, Disease prevention, Health care, Health occupations

## Abstract

This study aims to investigate whether wearing a filtering facepiece class 3 respirators with personal protective equipment (FPP3/PPE) during work in the intensive care unit (ICU) affects the blood saturation (SpO2), the heart rate (HR), and the well-being of health care workers (HCWs). This preliminary study included a group of 21 volunteers (including 16 females (76%), with a median age of 23 years). Each worker served as his own control and performed the test two times: they wore the FFP3/PPE and did not wear it for a three-hour shift in the ICU. The working with an FFP3/PPE compared to not working with an FFP3/PPE caused a significant, but within normal ranges, influence on the level of SpO2 with a mean decrease of − 1.43%. The highest reduction in the SpO2 was − 2.29% and occurred after 150 min of work. All of the score scales of the well-being markers increased consecutively but moderately during the shift while wearing the FFP3/PPE. We assume that a 3-h shift rhythm is a safe and reliable solution, i.e., three hours of working in the FFP3/PPE in the ICU, followed by rest or working without an FFP3/PPE.

## Introduction

Healthcare workers (HCWs) who take care of COVID-19 patients are subjected to the direct exposure to the virus. It is crucial during the COVID-19 pandemic that we should be focused on the intensive care unit staff who create the main and the last line of defense in COVID-19. Therefore, during the COVID-19 pandemic, there has been greater attention given to personal protective equipment (PPE). It comprises of the single-use disposable plastic repellent aprons or gowns with helmets or medical hood covers, goggles, and masks; the PPE must be worn by all HCWs working in intensive care units during the COVID-19 pandemic. The PPE could influence on HCWs' health^1,2^. For example, the gowns could impair cutaneous respiration, could cause an increase in sweating, and increase the feeling of discomfort. Several recent papers support the effectiveness of face masks in the COVID-19 pandemic in terms of protecting both HCWs and patients^1–6^. Talking or coughing generates both respiratory droplets, aerosolization, and liquid splashing. Therefore, filtering facepiece class 3 (FFP3) respirators are recommended for aerosol-generated procedures while providing a reasonable protection from COVID-19 and other viral infections^6,7^. According to the US National Institute for Occupational Safety and Health classification, FFP 3 respirators (EN149:2001. A1:2009) have a filter efficiency of 99% inward and a leakage of 2%, as well as have a reliable resistance against patients with a SARS-CoV-2 infection^7–10^. The “95” designation means that the N95 respirator blocks at least 95% particles (and FFP2 blocks 94%), and FFP1 respirators demonstrated only about 80% bacterial filtration efficiency of very small (0.3 μm) test particles^8–12^. The pandemic has brought unprecedented problems because the widespread use of PPEs carries risk for both environmental and human health. Nowadays, many used PPEs are being discarded, and therefore plastic waste pollution has grown exponentially. Thus, it causes increasing ecological degradation.

Another related (and still not fully recognized) problem is the health threats of HCPs associated with wearing older types of PPE. While in the manufacturing process, some chemicals could be added to PPE intentionally or even unintentionally. It needs further studies^13,14^. For this reason, novel materials of PPE have attracted significant attention. Highly advanced materials for personal thermal and moisture management are being launched^14^. The resistance to water without eliminating the permeability of the protective layer could be achieved by using advanced solutions of PPE systems. The most promising technologies include Janus textiles with the unidirectional water transport property; infrared-transparent visible-opaque (ITVO) fabrics; and nonwoven fabrics called melt-spun non-wovens; and thermal conductive textiles, as well as thermoelectric modules that deliver a steady and sustainable cooling effect through thermal contact with human skin^14^.

As far as masks are concerned, there were efforts to apply auxiliary components into face masks to enrich the functionalities and improve the daily monitoring and early warning of potential danger. Therefore, (bio)sensors are embedded in the masks, which serve as effective support for passive personal protection. Another achievement is a self-disinfecting mask which could be purified by built-in high voltage, and others^11,14,15^

While most of the recent studies concern FFP2 respirators, surprisingly few reports have shown the influence of wearing FFP3 respirators on the blood saturation and the well-being of HCWs working in hospitals' intensive care units (ICU) or COVID-19 departments^16,17^. One of the hypotheses assumes that masks can increase resistance to inspiration and respiration, which could decrease the efficacy of breathing^18^. The problem concerns intensive care specialist doctors and nurses who often spend long hours in full PPE with FFP3 respirators in emergency or COVID-19 dedicated departments.

This study aims to investigate whether wearing the face masks, FFP3 and PPE (FPP3/PPE), while working in the ICU affects the blood saturation, the heart rate (HR), and the well-being of HCWs.

We attempt to reject the null hypothesis that there is no relation between wearing FFP3/PPE and the blood saturation, heart rate, and well-being of HCPs.

## Methods

### Study time and participants

The study was performed in February and March 2021 in the Department of Anesthesiology and Intensive Therapy (DAIT) of Poznan University of Medical Sciences. All the HCWs working on the day shifts in the DAIT during the third peak of the pandemic were asked to take part in the study, and 21 of them agreed. Therefore, the study included a group of 21 volunteers (including 16 females (76%) and five males), with a median age of 23 years, with interquartile ranges (IQRs) of the participants (21–27), range of 20–59.

The study group consisted of intensive care registered nurses (RN, MSc or RN, BSc) (n = 3), nursing students (of second and third years of study) (n = 14), medical students (fifth year) (n = 3), and a senior consultant (MD, PhD) of the department. All of the students participating in the experiment were students in our university, and all were Caucasian.

All participants had valid medical examinations and were allowed to work by the recognized occupational doctor, and all were vaccinated twice with the COVID-19 vaccine (Pfizer/BioNTech). Persons with a history of pulmonary disease, anemia, vascular diseases, and other diseases that could limit respiratory function were excluded from the study.

### Research design

To evaluate the effects of wearing the respirators, we used a counterbalanced crossover design—a self-controlled trial. We wanted to rule out the possibility of natural changes in the pulse oximetry (SpO2) and heart rate parameters during the normal functioning of the participants. Therefore, each subject served as his own control and performed the test two times: (1) without a face mask and PPE (control) for three hours and (2) wearing an FFP3 respirator with full PPE for a 3-h shift in the intensive care unit. The first measurements ("time point 0") of the SpO2 and pulse rate were recorded 2 min after fitting the FFP3/PPE to give participants time to adapt themselves.

The study was performed during the third peak of the COVID-19 pandemic. Although the department was not an isolation ward dedicated only to COVID-19 patients, all emergency admissions were made before obtaining the test for a SARS-CoV-2 infection. Therefore, the applicable unit protocol required the team to wear the PPE and masks. During the test runs without masks and PPE, we encouraged the participants to perform each hour's same exercises for at least 10 min with the supervision of the study coordinator (IW).

**Figure 1:**
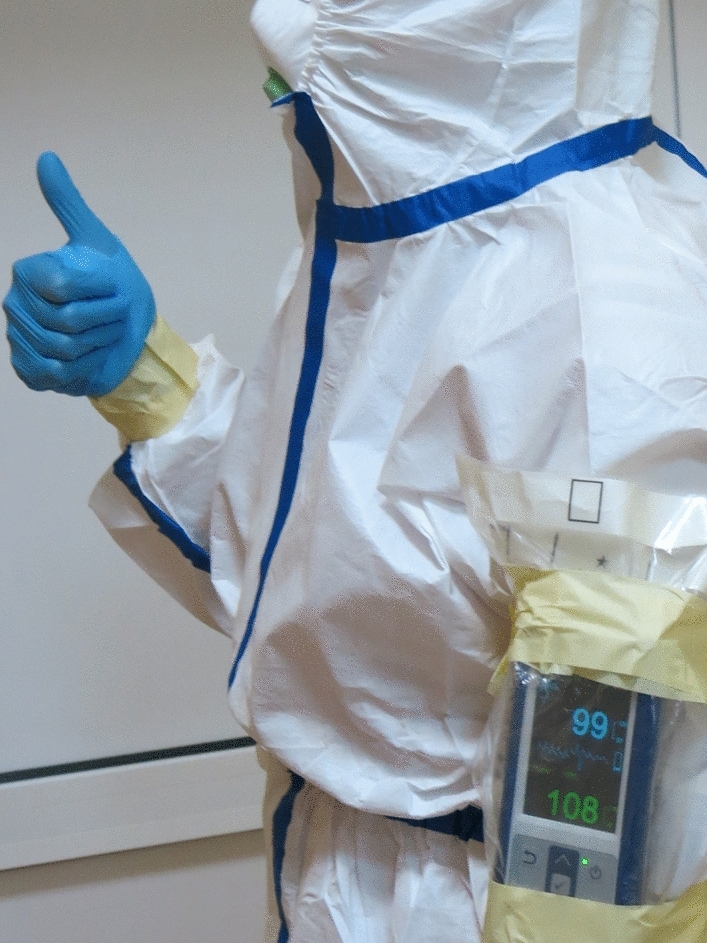
Nellcor PM 10 N monitoring system on a volunteer's hand. Such localization for safety and controlling reasons allowed the study coordinator to assess the recorded parameters in real time.

Among the study participants, we had students; therefore, for safety and controlling reasons, we used the Nellcor PM10N monitoring system, which allows the study coordinator to assess the recorded parameters in real time (Fig. 1).

To avoid bias, each subject's sequence of interventions (test run or shift run) were randomly assigned. Strenuous physical activity was prohibited during the 24 h preceding the test, and a night sleep of at least 6 h was mandated^17^. We also asked the participants not to use any fingernail polish.

### Equipment

Each volunteer was wearing the same type of respirators and PPE. We used FFP3 masks—(Oxy-line respirators: X310 SV FFP3 RD, CE1437. EN149:2001 + A1:2009, PPE, made in Poland) with gowns (Disposal Medical Integrated Protective Coverall. RAEX. Hunan Xunzhuo Industry, made in China), and goggles (made in Poland).

Blood saturation and HR were measured in real time for the duration of the whole testing with a pulse oximeter (Nellcor—Portable SpO2 Patient Monitoring System. PM10N, Coviden. Mansfield USA, made in Korea) (Fig. 1). The single-use SpO2 sensor (Nellcor. Neonatal-Adult SpO2 Sensor. MAXIN. Coviden. Mansfield USA, made in Mexico) was kept under the surgical glove on the fifth finger of the nondominant hand and connected under the gown with a PM10N device.

Additionally, every 30 min during the three-hour shift and during the control run, each subject completed a questionnaire concerning their well-being, with a score scale (ranging from 0 (no symptoms) to 6 points (unable to work)) for headache, shortness of breath, perspiration, fatigue, and thirst (see Table 1). More details about the score were provided in the Supplemental materials (Supplement 1).

**Table 1:** The results of blood saturation and heart rate in HCWs wearing the FFP3/PPE with a breakdown based on sex and age.

Results according to gender					
Variable	Female	Male	p-value	Median difference	95%CI
SpO2 (%) [median. IQR*]					
With FFP3/PPE	98 (97–99)	97 (96–98)	< 0.001	0.999	0.999313 – 1.000051
Without FFP3/PPE	99 (98–100)	97 (96–98)	< 0.001	1.999974	1.99995–1.99997
**Pulse beats per minute (HR) [median. IQR]**					
With FFP3/PPE	110 (99–119)	87 (77–93)	< 0.001	22.00005	22.00005–23.00004
Without FFP3/PPE	77 (70–85)	75 (71–79)	< 0.001	2.000064	1.999942–2.000006

Results according to age					
Variable	Age below 35	Age equal or over 35	p-value	Median difference	95%CI
**SpO2 (%) [median. IQR]**					
With FFP3/PPE	97 (96–98)	96 (95–97)	< 0.001	1.000068	1.000046–1.000068
Without FFP3/PPE	99 (98–100)	97 (96–97)	< 0.001	1.99998	1.99998–1.99993
**Pulse beats per minute (HR) [median. IQR]**					
With FFP3/PPE	105 (92–117)	93 (81–116)	< 0.001	6.999953	6.000032–7.000004
Without FFP3/PPE	76 (70–83)	80 (75–84)	< 0.001	3.00008	3.999951–2.999947

*Interquartile range.

### Statistical methods

To analyze the results of the SpO2 and the HR in the HCWs wearing the FFP3/PPE, the patients were grouped according to sex and age, and the median values along with the rest of the quartiles were reported (Table 1). All pairwise comparisons were made using the Mann–Whitney U-test^19^. Due to the number of all the measurements over time, we expected statistically significant differences between the medians. To measure the differences between the groups, we calculated the 95% confidence intervals or interquartile range for the differences of the medians.

Tables 2 and 3 present the results of the linear mixed-effects model (LMM) estimating the influence of working with the FFP3/PPE in the HCWs, the demographic factors and the levels of SpO2 [%]. LMM is a generalization of the standard linear model used, as the measurements are permitted to exhibit correlation and nonconstant variability between the individuals. LMM, therefore, provides the flexibility of modeling not only with the means but also with their variances and covariances. Moreover, LMM is recognized as a type of method that has some crucial advantages over repeated-measures ANOVA^20^.

**Table 2:** The model showed the influence of working in the FFP3/PPE for the SpO2, taking into consideration both age and gender.

	Coefficient	2.5%	97.5%	p-value
Intercept	98.44	97.67	99.22	**< 0.001**
FFP/PPE: yes	− 1.42	− 1.43	− 1.41	**< 0.001**
Sex: male	0.33	− 0.38	1.05	0.378
Age	− 0.02	− 0.05	0.01	0.185

In such cases, our model has the following form: SpO2[%] ~ working in FFP3/PPE + Sex + Age + (Time/Person).

Significant values are bold.

**Table 3:** Linear mixed-effects model (LMM): SpO2 [%] ~ FFP3/PPE + FFP3/PPE: time + (Second/person).

FFP3/PPE SpO2 status	Coefficient [%]	2.5%	97.5%	p-value
(Intercept)	98.193	97.582	98.804	< 0.001
FFP3/PPE: with FFP3/PPE: Time00:02:00	**0.476**	− 0.171	1.123	**0.151**
FFP3/PPE: without FFP3/PPE:Time 00:30:00	0.085	− 0.581	0.751	0.802
FFP3/PPE: with FFP3/PPE:Time 00:30:00	− **1.429**	− 2.076	− 0.781	** < 0.001**
FFP3/PPE: without FFP3/PPE:Time 01:00:00	0.54	− 0.162	1.242	0.133
FFP3/PPE: with FFP3/PPE:Time 01:00:00	**− 1.762**	− 2.411	− 1.114	** < 0.001**
FFP3/PPE: without FFP3/PPE:Time 01:30:00	0.461	− 0.289	1.211	0.23
FFP3/PPE: with FFP3/PPE:Time 01:30:00	**− 2.001**	− 2.651	− 1.35	** < 0.001**
FFP3/PPE: without FFP3/PPE:Time 02:00:00	− 0.18	− 0.931	0.572	0.64
FFP3/PPE: with FFP3/PPE:Time 02:00:00	**− 2.286**	− 2.939	− 1.633	** < 0.001**
FFP3/PPE: without FFP3/PPE:Time 02:30:00	0.584	− 0.211	1.379	0.152
FFP3/PPE: with FFP3/PPE:Time 02:30:00	**− 2.191**	− 2.848	− 1.535	** < 0.001**
FFP3/PPE: without FFP3/PPE:Time 03:00:00	1.083	− 0.501	2.667	0.182
FFP3/PPE: with FFP3/PPE:Time 03:00:00	**− 1.667**	− 2.345	− 0.989	** < 0.001**

Significant values are bold.

Table 4 presents a comparison of the different well-being markers between the 30-min time points while working in the FFP3/PPE versus not working in the FFP3/PPE. In such cases, Friedman's one-way repeated measures analysis of variance by ranks (Friedman test)^21^ was used to estimate the differences between consecutive groups (i.e., experiencing various ailments after 30, 60, 90, 120, 150, and 180 min).

**Table 4:** Comparison of the different well-being markers between the 30-min time points and while working in the FFP3/PPE (n = 21).

Factor	Averages	30 min	60 min	90 min	120 min	150 min	180 min	p-value*
Shortness of breath (dyspnea)	Mean (SD)	*0.33 (0.58)*	*0.43 (0.6)*	*0.57 (0.75)*	*0.57 (0.75)*	*0.76 (0.89)*	*0.86 (0.96)*	** < 0.001**
	Median (IQR)	**0 (0–1)**	**0 (0–1)**	**0 (0–1)**	**0 (0–1)**	**1 (0–1)**	**1 (0–1)**	
	Range	0—2	0—2	0—2	0—2	0—3	0—3	
Fatigue	Mean (SD)	*0.19 (0.4)*	*0.43 (0.51)*	*1 (0.77)*	*1.19 (0.81)*	*1.52 (0.93)*	*1.76 (0.83)*	** < 0.001**
	Median (IQR)	**0 (0–0)**	**0 (0–1)**	**1 (0–2)**	**1 (1–2)**	**1 (1–2)**	**2 (1–2)**	
	Range	0—1	0—1	0—2	0—3	0—3	1—3	
Thirst	Mean (SD)	*0.19 (0.51)*	*0.62 (0.8)*	*0.95 (1.02)*	*1.43 (1.12)*	*1.86 (1.06)*	*2.1 (1.26)*	** < 0.001**
	Median (IQR)	**0 (0–0)**	**0 (0–1)**	**1 (0–2)**	**2 (0–2)**	**2 (1–2)**	**2 (2–3)**	
	Range	0—2	0—2	0—3	0—3	0—4	0—5	
Headache	Mean (SD)	*0.05 (0.22)*	*0.14 (0.48)*	*0.29 (0.56)*	*0.29 (0.72)*	*0.52 (0.98)*	*0.67 (1.02)*	** < 0.001**
	Median (IQR)	**0 (0–0)**	**0 (0–0)**	**0 (0–0)**	**0 (0–0)**	**0 (0–1)**	**0 (0–1)**	
	Range	0—1	0—2	0—2	0—3	0—3	0—3	
Perspiration (sweating)	Mean (SD)	*0.62 (0.8)*	*0.9 (0.7)*	*1.48 (0.81)*	*1.67 (0.66)*	*2 (0.84)*	*2.19 (0.93)*	** < 0.001**
	Median (IQR)	**0 (0–1)**	**1 (0–1)**	**2 (1–2)**	**2 (2–2)**	**2 (2–3)**	**2 (2–3)**	
	Range	0—2	0—2	0—3	0—2	0—3	0—3	

Results depicted in scoring scale (score range 0 to 6, see also Supplemental materials 1 for details).

Significant values are in bold and italics.

*Assessed by Friedman’s test.

Statistical analysis was performed using RStudio (Version 1.3.1073).

### Ethics approval

The study was conducted according to the Helsinki Declaration (Seventh revision) and Good Clinical Practice Guidelines^22^. The study protocol was accepted by the Bioethics Committee of Poznań University of Medical Sciences, Poland (Ref. no.:123/21 on 04th Feb. 2021) and each participant gave informed consent. Participants were informed of the anonymous data extraction and analysis from the study files.

### Consent for publication

All authors have given their consent for publication.

## Results

We obtained 404,195 measurements of blood saturations (SpO2) in general. There were 156,113 and 248,082 measurements without and with the FFP3/PPE, respectively.

The median SpO2 without and with the FFP3/PPE was 99% (IQR 98–100%) and 97% (IQR 96–98%), respectively. The difference was significant (p < 0.001).

The median number of pulse beats per minute (HR) without and with the FFP3/PPE was 76 (IQR 71–84) and 104 (IQR 91–117), respectively, and the difference was also significant (p < 0.001). The mean results of the SpO2 and HR for the whole shift duration of the HCWs in the FFP3/PPE and without the FFP3/PPE with a breakdown based on sex and age category are shown in Table 1. Men generally had a lower SpO2 (by 1%) and a lower pulse rate (by 22 PB/min) while wearing the FPP3/PPE than women and also had a lower SpO2 (by 2%) and a lower pulse rate (by 2 PB/min) when not wearing the FFP3/PPE. The older group showed a slightly lower SpO2 and HR when wearing the FFP3/PPE and when not wearing the FFP3/PPE and had a slightly higher pulse rate without the FFP3/PPE than the younger group (Table 1).

The model demonstrated the influence of working in the FFP3/PPE on the SpO2 when sex and gender were considered, and results are shown in Table 2.

The reduction of the standard deviation measured from this model due to the variability between the measurements of the individual features was equal to 32.4%. This means that working in the FFP3/PPE had a significant influence on the SpO2, and it was associated with an average decrease in the SpO2 by 1.42% (p < 0.001) in comparison to measurements done without this kind of PPE. Additionally, we developed a mixed-effects model that considered the fact of wearing the FFP3 in an interaction with the time of measurements (periods of each consecutive 30 min).

**Figure 2:**
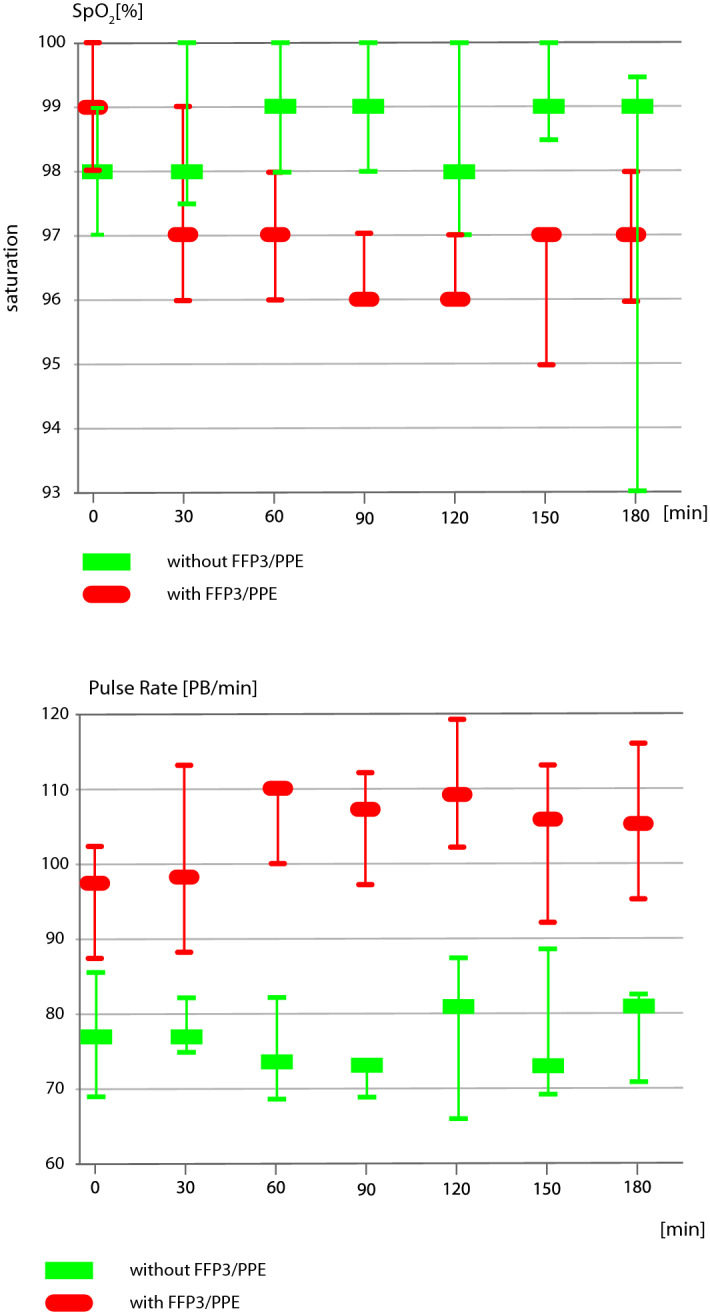
Changes in the SpO2 and the heart rate (PB/min) in the HCWs working in the FFP/PPE and without the FFP/PPE throughout the whole shift time (medians and interquartile range are shown).

The mixed model demonstrated that HCWs wearing the FFP3/PPE compared to the period than did not work in the FFP3/PPE had a significant influence on the levels of SpO2. A decrease in the SpO2 was observed after 30 min from the start of work, and the highest reduction in the SpO2 (− 2.29%) occurred after 120 min, and after 150 min, the decrease was − 2.19%. At 3 h, we noticed a decrease in the SpO2 of − 1.68%, and the mean decrease in the SpO2 within a 3-h shift was − 1.43%. We also found a significant increase in the HR after 60, 90, and 120 min from the start of work to 18, 8.5, and 13 PB/min., respectively. (The LMM, which investigates the influence of wearing the FFP3/PPE on the HR along with the interaction effect on the time of measurement, is shown in the supplementary materials, see Supplement 2 for details). The changes in the SpO2 and HR during the 30-min episodes throughout the study time are depicted in Fig. 2.

All of the score scales of the well-being markers increased consecutively with the time spent working in the FFP3/PPE (see Table 4). The most notable differences between the beginning and the last 30 min of a shift were observed for fatigue, thirst, and sweating. For all of the parameters, the median score of the scale was 2 (out of a maximum score of 6). There was no score checked during test runs without the FFP3/PPE, where the median score was taken as a 0.

Additionally, we did not find any differences between women and men or between younger and older (> 35 years) HCWs in terms of the results of the above scoring scale that depicted the well-being of the HCWs.

## Discussion

In our study, SpO2 and HR were measured continuously (we collected over 400,000 measurements for all 21 participants). This allowed us to construct more reliable mixed model formulas.

The main finding of this study was that working in an FFP3/PPE caused a moderate but significant decrease in the SpO2. The median decrease in the SpO2 within 3 h of the shift was approximately 2% to 3% (dropping from 99 to 97% or 96%, depending on the time after the shift start), but this did not reach the levels that are recognized as dangerous for health. We also found that the HR increased from 77 to 110 pulse beats/minute (PB/min) within the same periods in the shifts. The decrease in the SpO2 could be observed after the first 30 min of a shift. The difference was significant until the end of shift, i.e., after 150–180 min of working. However, after a significant drop in the SpO2 within the first period (2–30 min), the following 30-min consecutive periods did not differ significantly, demonstrating a stable decrease in this parameter (see Fig. 2).

To a lesser extent, we observed an increase in the HR. It was noticed to be the most prominent by 13 to 18 PB/min, compared the first 30-min period to the periods between 60 and 120 min of the shift (see Fig. 2).

We found lower SpO2 and pulse rates in men than in women (Table 1). Normal SpO2 readings usually range from 95 to 100%^23^. Values under 90% are considered low, and values of 92% are sufficient to predict adequate oxygenation in Caucasian persons (a saturation of 95% is usually required in persons of African descent)^24^. Therefore, the obtained results do not exceed the level that is considered safe. Similarly, the self-assessed shortness of breath was relatively mild as a median score for the first 120 min of the shift was 0, and in the last 60 min, the score was 1. The most severe problems associated with PPE and impermeable materials, as thirst and perspiration were the most prominent ailments—for both, the median scores were 2 in the last 30 min of the shift.

Regarding the question of how wearing masks influence the SpO2, there is high heterogeneity in the literature. Different kinds of masks were tested under different experimental conditions^16–18,25–28^. Beder et al. obtained similar results while studying disposable sterile one-way surgical paper face masks and revealed a consecutive decrease in the SpO2 during the 2 and 3 h of surgical operations (drop by 1% and 1.5%, respectively) and a slight increase in HR compared to preoperative values in the surgeons. The decrease in the saturation was more prominent in surgeons aged over 35^25^.

Recently, Fantin performed an arterial blood gas analysis in a 30-year-old female resident, which was carried out during a 13-h day shift on the COVID-19 Intensive Care Unit. This study found that during the multihour wearing of FFP3 masks, all measured values were within the normal ranges, although a trend toward an insufficient gas exchange could be seen. She had an increase in the partial pressure of carbon dioxide (PaCO2), from 29.3 to 36.7 mmHg, showing a continuous decrease during the shift in the partial pressure of oxygen (PaO2), from 102 to 88.8 mmHg. The changes could be seen within the first five hours of the trial; unfortunately, the study consisted of just one young volunteer. Therefore, no further conclusions can be drawn from the study^17^.

In another study, 20 young (mean age of 29 years) oral surgeons were enrolled. The SpO2 was measured twice, before and after oral surgeries were performed. They used disposable sterile one-way surgical paper masks and FFP2 masks combined, which could not be formally treated as a reliable substitute for FFP3 masks. Nevertheless, the results corresponded with ours. In all 20 surgeons wearing the FFP2 respirators covered by surgical masks, a reduction in the SpO2 from approximately 97.5% before surgery to 94% after surgery was noted. Additionally, before surgery, the mean HR was 60 ± 9 beats/min, and after surgery, the dentists' HR increased to 98 ± 12 beats/min . Shortness of breath and light headedness/headaches were also noted. The durations of most of the operations were between 20 and 120 min (the shortest one was less than 20 min, and the longest was up to 240 min).

The main difference between our methodology and the study performed in the oral surgeons was that the SpO2 and HR were measured twice at the beginning and after the surgery. Therefore, we cannot exclude any fluctuations of the SpO2 and HR levels during surgery^16^. Another recent review demonstrated that face masks, including N95 respirators, surgical masks, and cloth face masks, may have some but relatively small and often difficult-to-detect effects on the work of breathing (which could cause dyspnea), blood gases, and other physiological parameters during physical activity, even with heavy exercise^26–28^.

Our model is easy to repeat and to perform comparative tests but has some shortcomings. Our study results should be considered preliminary. In the Department where the study was conducted, we have a rather young team of HCWs. We did not find any important, from a clinical point of view, differences between the older and younger HCWs (HCWs over 35 years of age had their SpO2 drop from 97 to 96%, while the younger HCWs had their SpO2 drop from 99 to 97% without and with the FFP3/PPE, respectively (see Table 1 for details)). Therefore, it seems that with regard to the young and middle-aged HCWs, there were no essential differences of the assessed parameters between sex and age groups. However, we cannot exclude the possibility that a higher number of participants at an older age, especially those over 50 or 60 years of age, could have different results.

The next problem is that we performed this study during only one 3-h shift. However, another “real world” study, which evaluated staff wearing the PPF2/PPE and working in the COVID-19 Isolation Ward, demonstrated that during the next 3 h of a shift (after 3 h of rest), the results of physiological parameters and well-being score scales did not differ from the first shifts. Therefore, we can assume that 3 h of rest between 3 h of shifts was sufficient to regenerate the health of the staff members, while taking into consideration a 12-h shift (with the pattern: 3 h of working-3 rest-3 working-3 rest, (unpublished data)).

However, we should take into consideration that HCWs have been working in shifts throughout the whole pandemic period that has now exceeded two years. Therefore, to avoid burn-out and chronic fatigue syndromes, we should seek to ensure as much comfort as possible while working^29–31^. In particular, rules should be taken to avoid extensive working hours, to ensure the provision of advanced equipment—especially more comfortable gowns and respirators—and to maintain a high level of occupational safety, health care, proper social conditions and compliance for the rules of the law and other health and safety requirements. Some countermeasures to avoid health risks of HCWs have been take and has already been discussed in the introduction. Additionally, we should bear in mind that any of the disadvantages connected with wearing an FFP3/PPE should be considered in the context of the significant reduction of the risks of contracting a SARS-CoV-2 infection by HCWs and, consequently significantly decreasing the risk of the transfer of infection to our patients in hospital wards^1–6^.

## Conclusions

We note that although the changes of parameters were statistically significant during the shift, they did not exceed the norm. Despite small physiological changes, most HCWs complained about the moderate discomfort of working in an FFP3/PPE, and they mainly complained about fatigue, thirst, and perspiration. We did not find any differences in the well-being parameters between the gender or age groups; additionally, there was no correlation between the SpO2 and HR and the well-being factors in the study workplace.

We assume a 3-h shift rhythm –, i.e., three hours of working in the FFP3/PPE in the isolation ward (red zone), followed by rest or working without an FFP3/PPE (in positions that do not require them) is a safe and reliable solution.

## Supplementary Information

Supplementary Information 1.

Supplementary Information 2.

## Data Availability

Data and materials are available on request to the corresponding authors.
