# Supplementary material for: The impact of FFP3 respirators on the blood saturation
Source: Sci Rep. 2022 Jan 25;12:1335. doi: 10.1038/s41598-022-05319-3 (PMC8789906; doi:10.1038/s41598-022-05319-3)
Supplement: Supplementary file 1 — Supplementary Information 1. [file 41598_2022_5319_MOESM1_ESM.docx]

The impact of FFP3 respirators on the blood saturation.

Wojtasz I, Jaracz K, Sobczynski P, Druzdz A, Dyk D, and Kazmierski R.

SUPPLEMENT 1

The score scale, a tool for self-assessment of Health Care Workers well-being

The score scale (ranging from 0 to 6 points) for the mentioned below symptoms:

headache,

shortness of breath,

perspiration,

fatigue,

and thirst

Score scale:

0. No symptoms

1. Small symptoms, acceptable, non-disruptive work

2. Mediocre symptoms, non-disruptive work

3. Moderate symptoms, with slight work disruption

4. Burdensome working conditions that disrupt work

5. Severe symptoms that considerably disrupt work

6. Symptoms forced the work to be broken off (Please, give an exact interruption time)
