# Supplementary material for: The impact of FFP3 respirators on the blood saturation
Source: Sci Rep. 2022 Jan 25;12:1335. doi: 10.1038/s41598-022-05319-3 (PMC8789906; doi:10.1038/s41598-022-05319-3)
Supplement: Supplementary file 2 — Supplementary Information 2. [file 41598_2022_5319_MOESM2_ESM.docx]

The impact of FFP3 respirators on the blood saturation.

Wojtasz I, Jaracz K, Sobczynski P, Druzdz A, Dyk D, and Kazmierski R.Supplementary materials.

SUPPLEMENT 2.

Table 1 S.

Linear mixed-effects model (LMM):

***HR (pulse beats/minute)*** *~ FFP3/PPE + FFP3/PPE: time + (Second/person)*

| **FFP3/PPE HR Status** | Coefficent  [HR] | 2.5 % | 97.5 % | p-value |
| --- | --- | --- | --- | --- |
| **(Intercept)** | **77,28** | 69,845 | 84,715 | **<0,001** |
| **FFP3/PPE: with FFP3/PPE: Time 00:02:00** | **18,333** | 10,158 | 26,508 | **<0,001** |
| **FFP3/PPE: without FFP3/PPE:Time 00:30:00** | -1,317 | -9,738 | 7,105 | 0,76 |
| **FFP3/PPE: with FFP3/PPE:Time 00:30:00** | 4,957 | -3,234 | 13,148 | 0,237 |
| **FFP3/PPE: without FFP3/PPE:Time 01:00:00** | -2,705 | -11,611 | 6,2 | 0,552 |
| **FFP3/PPE: with FFP3/PPE:Time 01:00:00** | **18,2** | 9,962 | 26,437 | **<0,001** |
| **FFP3/PPE: without FFP3/PPE:Time 01:30:00** | -8,401 | -17,983 | 1,182 | 0,087 |
| **FFP3/PPE: with FFP3/PPE:Time 01:30:00** | **8,536** | 0,222 | 16,851 | **0,046** |
| **FFP3/PPE: without FFP3/PPE:Time 02:00:00** | -3,338 | -13,026 | 6,351 | 0,5 |
| **FFP3/PPE: with FFP3/PPE:Time 02:00:00** | **13,207** | 4,786 | 21,629 | **0,002** |
| **FFP3/PPE: without FFP3/PPE:Time 02:30:00** | -3,709 | -14,071 | 6,652 | 0,484 |
| **FFP3/PPE: with FFP3/PPE:Time 02:30:00** | 8,402 | -0,155 | 16,959 | 0,057 |
| **FFP3/PPE: without FFP3/PPE:Time 03:00:00** | 1,805 | -18,941 | 22,551 | 0,865 |
| **FFP3/PPE: with FFP3/PPE:Time 03:00:00** | 7,437 | -1,521 | 16,396 | 0,108 |
